# Validation of the Chinese version of the coping strategies for victims of cyberbullying scale

**DOI:** 10.1186/s40359-024-01766-x

**Published:** 2024-05-10

**Authors:** Qiqi CHEN, Zujian LU, Bofan LIU, Qiao XIAO, Yuhong ZHU, Ko Ling CHAN

**Affiliations:** 1 Department of Applied Social Sciences, The Hong Kong Polytechnic University, Hung Hom, Hong Kong, China; 2 Department of Social Work, School of Sociology and Anthropology, Xiamen University, Xiamen, Xiamen, China; 3 Department of Social Work & Social Policy, Renmin University of China, Beijing, China

**Keywords:** Validation, Cyberbullying coping, Cyberbullying questionnaire, Chinese adolescents, Sexual and gender minorities

## Abstract

**Background:**

Although abundant evidence has confirmed cyberbullying as a global online risk, little is known about the coping strategies employed by victims and those who experiencing bullying. A validated scale for coping with cyberbullying could inform evidence-based social services and enable comparative studies of this phenomenon among victims from different backgrounds. This study aims to validate the Coping Strategies for Victims of Cyberbullying (CSVC) scale among Chinese adolescents and to compare its effectiveness between victims and bully-victims (individuals with dual roles).

**Methods:**

A 25-item CSVC scale was translated and adapted for cultural relevance in the Chinese context. A sample of 1,716 adolescents, aged 13–18 years, from two middle schools and one high school in China, was recruited. Both exploratory factor analysis (EFA) and confirmatory factor analysis (CFA) were conducted.

**Results:**

The EFA revealed that the Chinese version of the CSVC scale had satisfactory validity. The CFA demonstrated a good fit for the eight-factor model in assessing different coping strategies for cyberbullying. Differences in the selection of coping strategies were observed between the general adolescent population and sexual and gender minorities.

**Conclusions:**

Future intervention studies may use this validated scale to educate adolescents, both those affected by cyberbullying and those who are not, to learn a broader range of coping strategies and to choose more effective ones.

**Supplementary Information:**

The online version contains supplementary material available at 10.1186/s40359-024-01766-x.

## Background

The increasing development of cyberspace provides adolescents with opportunities for socialization and personal development, as well as associated risks such as cyberbullying [1]. Cyberbullying is broadly defined as aggressive behavior that deliberately inflicts harm or discomfort on others through digital media or electronic technology [2, 3]. Estimates of cyberbullying victimization in Chinese societies, including mainland China, Hong Kong, and Taiwan, suggest that around 30% of the total population of children, adolescents, and young adults are affected [4, 5]. Cyberbullying victimization has been correlated with behavioral problems, depressive symptoms, substance and alcohol abuse, and family-related factors such as frequent residential mobility [6, 7]. The unique nature of cyberspace has been identified as a special risk factor for cyberbullying. This includes the absence of spatial and temporal boundaries, the anonymity of users, and the ease of establishing interactions with strangers [8]. Studies have revealed that coping with cyberbullying can be more complex than coping with offline bullying [9]. Therefore, a special focus should be placed on coping strategies for cyberbullying, drawing on previously established models for coping with offline bullying.

Recent studies on cyberbullying have focused on coping strategies for cyberbullying victimization worldwide [4]. Coping often refers to the volitional efforts to regulate emotion, behavior, and cognition in order to deal with stressful events or situations [1]. Social information processing theory suggests that coping involves cognitive, emotional, and social processes that determine the appropriateness of responses [1]. Many researchers have investigated individuals’ coping with cyberbullying using two approaches: one that focuses on traditional coping strategies for stressful situations, including cyberbullying, and another that deals with cyber-specific technological solutions to cyberbullying [9]. For example, some scholars have applied traditional dichotomous strategies, such as emotion-focused versus problem-focused coping [10], and avoidance versus approach coping [11], based on the assumption that individuals’ responses to cyberbullying are generally consistent with their strategies for other stressful circumstances. However, other researchers have argued that traditional dichotomous models of coping with stress may not be adequately describe the complex situation in cyberspace [12]. Moreover, the prevalent approaches in cyberbullying, such as inaction, cannot be properly categorized into either of the classifications [9]. These cyber-specific coping strategies should be viewed as distinct categories that deserve more attention for the prevention of or intervention in cyberbullying.

Existing studies have identified strategies favored by cyberbullying victims, such as blocking the perpetrator on the website and seeking offline social support. Some of these studies have examined the effectiveness of these strategies [12]. Research has revealed that between 19% and 89.8% of adolescents experiencing cyberbullying victimization would seek help [13]. These strategies have been shown to help curb cyberbullying and buffer against adverse emotional effects, including depression, anxiety, and lowered self-esteem [14]. Conversely, lower levels of social anxiety and higher levels of self-esteem have been mentioned as determinants of effective coping [15]. Researchers have also identified preferred sources of support for victimized adolescents, including parents, peers, and teachers [13]. However, not all cyberbullying victims are proactive in seeking help; some tend to be avoidant, do nothing, self-blaming, retaliatory, or unable to control their emotions [16]. This reluctance to seek help has been attributed to fears of parental restrictions on digital devices or overconfidence in their ability to cope independently [4]. Studies have also reported that, in contrast to traditional bullying, cyberbullying victims rarely regard retaliation as a useful response and seldom employ it [12]. Further investigation into the individual factors that influence these coping styles is important for the development of effective cyberbullying interventions.

While it is clear that cyberbullying affects a wide range of adolescents, it is essential to recognize that some groups may experience it differently due to additional layers of vulnerability. Among these, sexual and gender minority (SGM) adolescents often face unique challenges that merit closer examination. Recent studies [2] have reported that SGM adolescents are among the most vulnerable populations to cyberbullying. This could be explained by the reproduction of homophobia and social discrimination on the Internet, which may lead to higher rates of victimization compared to their heterosexual counterparts [8]. Though many studies have been conducted on cyberbullying and its coping strategies in China, the evidence pertaining to SGM and coping with cyberbullying is inadequate; no programs have been found to specifically address cyberbullying among SGM [2]. Owing to the Confucian culture of family and intimate relationships, homosexuality is often regarded as a challenge to family obligations and the continuation of family bloodlines in China and other East Asian countries [17]. The motivations for cyberbullying, including fun, discrimination, jealousy, and revenge, are consistent with findings from Western research [18]. SGM, due to their differences in sexual orientation or identity, are often more vulnerable to being targeted by other students [17]. These findings underscore the pressing need to document the prevalence and nature of cyberbullying incidents among SGM during childhood and adolescence in Asian countries.

The continuation and negative effects of cyberbullying victimization appear to be related to victims’ coping strategies. However, investigations into and interventions for adolescents’ positive coping strategies are scarce and deserve further attention. Researchers often use self-report scales for victims to describe or report their coping responses. The most widely used include the Adolescent Coping Scale (ACS) [19] and the Self-Report Coping Measure (SRCM) [20], which are cost-effective and time-efficient methods for assessing the construct of coping. However, most existing scales are designed for general stressors rather than specifically for cyberbullying, which could be problematic; they may lack appropriate appraisals in a complex online environment. For example, online coping strategies could include actions such as saving evidence by taking a screenshot, closing a window, or changing privacy settings, actions that are not applicable to offline victimization [21]. Research on cyberbullying should consider the evolving nature of violence in cyberspace [22]. Therefore, the development of a psychometrically validated scale is of paramount importance for advancing knowledge of tailoring and implementing coping strategies for those experiencing cyberbullying victimization and for potential victims.

As reviewed above, understanding how victims cope with cyberbullying is crucial for developing effective interventions. In this study, we aim to adapt and validate the Chinese version of the Coping Strategies for Victims of Cyberbullying (CSVC) scale for adolescents. The questionnaire was originally developed by Machackova and colleagues [12]. We recognize the significance of this being the inaugural validation of the scale in a new linguistic and cultural context, which will serve as a credible tool for school professionals to design and evaluate individualized programs for cyberbullying victims. The validation of the scale among Chinese adolescents would contribute significantly to the research literature by providing a tool for future studies to compare cross-cultural differences in coping strategies and the effectiveness of various interventions. Additionally, we will use this scale to compare differences in coping strategies between the general adolescent population and SGM. We hypothesize that adolescent SGM might employ different coping strategies compared to their heterosexual counterparts.

## Methods

### Sample

This study used data from two middle schools in Qingdao and one high school in Wuhan, China, both classified as new first-tier cities. The participants were students in grades 8–12, aged 13–18 years, who were recruited between September and October 2022. This project aimed to explore the social determinants of adolescent cyberbullying and related health behaviors. Participants were invited to complete a web-based survey questionnaire at school. Informed consent was obtained from all students and their parents before conducting the study. Trained research assistants informed the participants that their involvement in the study would be anonymous and voluntary, and that they were free to withdraw at any time point during the survey. The questionnaire took approximately 30 min to complete. A sample of 1,716 adolescents returned valid questionnaires, of which 1,132 (66.0%) were middle school students and 584 (34.0%) were high school students. Approximately half of participants (44.52%, *n* = 764) were female. The average age of the participants was 14.60 years (SD = 1.35). The study protocol was approved by the institutional review boards of the universities with which the authors are affiliated.

### Measures

#### Translation of CSVC from English to Chinese

The items on the Coping Strategies for Victims of Cyberbullying (CSVC) scale were developed based on existing literature about coping strategies for cyberbullying [12]. The original CSVC comprises 26 items across nine factors: technological coping (6 items), reframing (4 items), ignoring (2 items), dissociation (4 items), cognitive avoidance (2 items), behavioral avoidance (3 items), seeking support (1 item), confrontation (2 items), and retaliation (2 items). Specifically, five factors: technological coping, retaliation, confrontation, seeking support, and avoidance, were identified based on the framework outlined by Perren et al. [23]. The authors expanded the scope of cognitive strategies, introducing the factor of dissociation, which pertains to the perceived incident in the online world from offline settings. This factor was hypothesized to act as a buffer against emotional harm [12]. Reframing was defined broadly as a positive appraisal by individuals who interpreted the incident as untroubling for reasons that were not bothersome.

A forward-backward method [24] was used to translate the original scale from English to Chinese, and then back to English, to compare the back-translated version with the original. The translation process was carried out by a panel of bilingual professionals proficient in both English and Chinese. This panel included social workers and graduate psychology students with research experience in interpersonal violence prevention. They possessed extensive experience in academic research translations, ensuring that the translated version preserved the original meaning. The professional panel was consulted again regarding the content of the translated scale [25]. For certain terms and phrases without direct equivalents in Chinese, the research team and the expert panel held discussions to address and reconcile any discrepancies, ensuring that the final version was conceptually equivalent to the original. The scoring method remained consistent with the original version, with all 26 items being dichotomous (0 = “no,” 1 = “yes”). The psychometric properties of the scale in the current study are described in the Results section.

#### Cyberbullying

We employed the Chinese version of the European Cyberbullying Intervention Project Questionnaire (ECIPQ) [26, 27] to measure participants’ cyberbullying experiences. The Chinese version of the ECIPQ comprises 14 items, divided into cyberbullying perpetration (7 items) and victimization (7 items). Example items include “I excluded or neglected someone in a social networking site” and “Someone sent or forwarded nasty things about me to others online.” Each item is rated on a 5-point Likert scale to assess the frequency of experiences in the preceding year (0 = “never,” 4 = “always”). Given that the CSVC scale was designed to measure coping strategies among cyberbullying victims, we recoded cyberbullying experiences into two categories: victimization, and perpetration-victimization. Specifically, participants who scored ≥ 1 on the victimization subscale and 0 on the perpetration subscale were coded as “Victimization.” Those who scored ≥ 1 on both the perpetration and victimization subscales were coded as “Perpetration-victimization.” The ECIPQ scale demonstrated good reliability in this study, with an overall Cronbach’s α of 0.96, and Cronbach’s α values of 0.95 for the perpetration subscale and 0.93 for the victimization subscale.

### Data analysis

To verify the reliability of the Chinese version of the CSVC scale [12], we used the original latent factor structure as the basis for our exploratory factor analysis (EFA) [28]. Corrected item-total correlations and variances in the explanatory variables were applied to determine the number of items to be retained. The internal consistency reliability was measured using Cronbach’s α. Confirmatory factor analysis (CFA) was performed to assess the fitness of the measurement model using the maximum likelihood method for estimation. First, we specified the latent and observed variables constituting the measurement model based on the EFA results. 25 out of the 26 items loaded onto the eight latent variables, with the exception of seeking support factor, which contained only one item. Second, a correlation coefficient was calculated for each latent variable, with each observed variable specified as having a measurement error. Chi-square statistics, root mean square error of approximation (RMSEA), comparative fit index (CFI), Tucker-Lewis index (TLI), and standardized root mean square residual (SRMR) were calculated. The following criteria were used to estimate the fitness of the measurement model: a non-significant chi-square goodness-of-fit value (*p* > .05) [29], RMSEA value ≤ 0.08 and the upper bound 90% confidence interval value ≤ 0.08, CFI and TLI values ≥ 0.90, and an SRMR value ≤ 0.08 [30]. The prevalence rates of coping strategies were summarized and compared based on different cyberbullying experiences, including victimization and perpetration-victimization. We also applied the coping strategy scores in a regression analysis to explore the differences between the general adolescent group and SGM. Participants who answered “yes” to the question “Have you ever experienced the above item because you were SGM” were categorized as experiencing “Cyberbullying victimization.” Those who answered “yes” to both the question “Have you ever experienced the above item because you were SGM” and “Have you ever perpetrate the above item to others because they were SGM” were categorized as experiencing “Cyberbullying perpetration-victimization.” All statistical analyses were conducted using SPSS version 20.0 (Chicago, IL, USA), and confirmatory factor analysis was conducted using Amos version 26.0 (Chicago, IL, USA).

## Results

### EFA result

Table 1 presents the exploratory factor analysis results for the Chinese CSVC scale. Most items demonstrated an explanation rate exceeding 50% in the extraction values, suggesting a robust factor structure for the Chinese CSVC [31]. The conventional cut-off value for corrected item-total correlations is set at 0.40, indicative of strong factor loadings [32]. Almost all items in this study met this criterion, with the exception of three items that showed coefficients between 0.36 and 0.38, still closely approaching strong loadings. The internal consistency reliability of the CSVC was calculated using Cronbach’s α, a measure indicating the coherence of a scale in assessing an underlying construct. The overall Cronbach’s α was 0.92, and the α coefficients for the eight factors were: Technological Coping (0.81), Reframing (0.84), Ignoring (0.91), Dissociation (0.86), Cognitive Avoidance (0.65), Behavioral Avoidance (0.67), Confrontation (0.78), and Retaliation (0.87). These results indicate satisfactory internal consistency for the Chinese CSVS, although the coefficients for Cognitive Avoidance and Behavioral Avoidance suggest potential areas for further investigation or refinement.

**Table 1:** Exploratory factor analysis results of the the CSVC scale

	Extraction	Corrected item-total correlations	Cronbach’s alpha
Total			0.92
Technological coping			0.81
1. Deleted my profile	0.67	0.44	
2. Changed my settings	0.79	0.48	
3. Deleted from contact list	0.73	0.52	
4. Changed my information	0.72	0.49	
5. Searched for advice	0.51	0.38	
6. Reported to administrator	0.64	0.46	
Reframing			0.84
7. Thought to myself not worth my time	0.62	0.57	
8. Thought to myself the person was pitiful	0.59	0.55	
9. Thought to myself that not hurt me	0.67	0.63	
10. Thought to myself nothing serious	0.73	0.64	
Ignoring			0.91
11. Decided to ignore it	0.73	0.63	
12. Not to pay attention	0.74	0.64	
Dissociation			0.86
13. Thought to myself it wasn’t happening in real life	0.66	0.66	
14. Thought to myself the person wouldn’t do it in real life	0.67	0.66	
15. Thought to myself things in real life like that would be much worse	0.38	0.54	
16. Thought to myself things similar happen on the internet	0.62	0.63	
Cognitive avoidance			0.65
17. Tried to focus on something else	0.58	0.64	
18. Simply took it lightly	0.57	0.58	
Behavioral avoidance			0.67
19. Stopped visiting the webpage	0.62	0.60	
20. Deleted the messages	0.60	0.61	
21. Started avoiding the person in real life	0.36	0.44	
Seeking support			/
22. Told someone about it	0.37	0.42	
Confrontation			0.78
23. Tried to talk to the person on the internet to persuade him or her to stop	0.60	0.46	
24. Tried face-to-face talking to persuade him or her to stop	0.60	0.45	
Retaliation			0.87
25. Did something similar face-to-face to that person	0.82	0.36	
26. Did something similar online to that person	0.80	0.36	

### CFA result

**Fig. 1:**
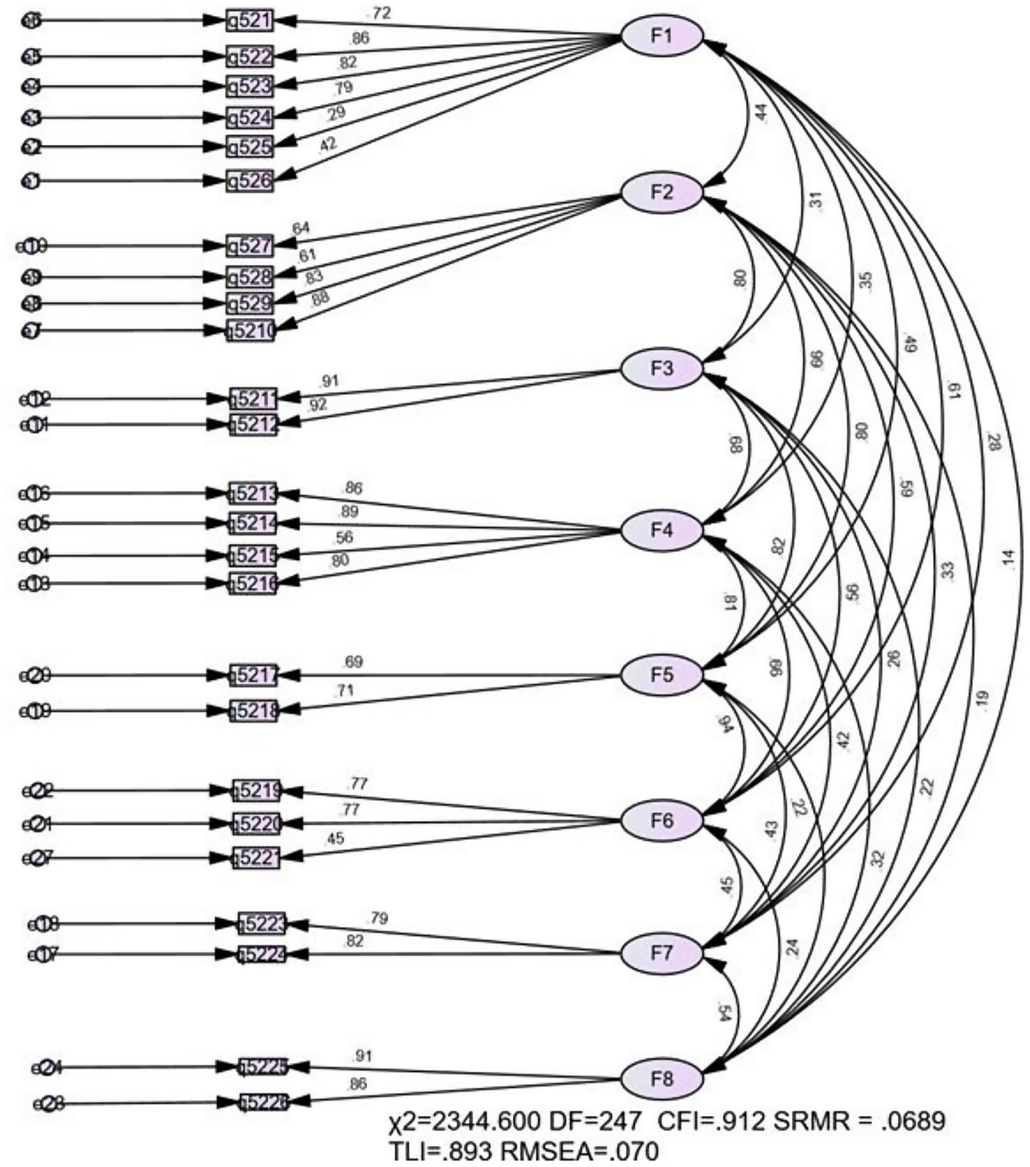
The eight-factor model of the CSVC scale. ***p < .001

Following the factor structure identified by the EFA, we maintained the original factor arrangement in the CSVC questionnaire [12], excluding the “seeking support” factor, which contained only one item. Consequently, an eight-factor model with 25 items was generated for the CFA. As shown in Fig. 1, the CFA results revealed a satisfactory fit of the measurement model. The model demonstrated the following indices: a χ^2^ value of 2344.60 (df = 247, *p* < .001), an RMSEA index of 0.070 (90% CI [0.068, 0.073]), a CFI of 0.912, a TLI of 0.893, and an SRMR of 0.070. All parameters for each factor were statistically significant (Table S1), and most factor loadings were robust, except for the three-factor loadings (all values > 0.60; *p* < .001), indicating good convergent validity [33]. Therefore, the CFA results support the eight-factor model as a good fit, providing preliminary evidence for the reliability and validity of the Chinese version of the CSVS.

### Prevalence rates of the Chinese CSVC items

We then applied the Chinese CSVC to compare coping strategies between those who experienced cyberbullying victimization and those with perpetration-victimization dual roles. As shown in Tables 2 and 610 (35.55%) participants reported having experienced cyberbullying victimization in their lifetime, and 208 (12.12%) reported both perpetration and victimization. Retaliation, which includes actions such as “Did something similar online or face-to-face to that person,” appeared to be the most commonly used coping strategy among the general population participants (78.03%) and cyberbullying victims (72.46%). For those who were both perpetrators and victims of cyberbullying, the most commonly used coping strategy was behavioral avoidance (66.83%), encompassing actions such as “Stopped visiting the webpage,” “Deleted the messages,” and “Started avoiding the person in real life.” However, seeking support was found to be the least used coping strategy in response to cyberbullying for all participants (38.24%), exclusively cyberbullying victims (41.64%), and perpetrator-victims (47.12%).

**Table 2:** Prevalence rates of the CSVC scale items by cyberbullying experiences

*N* (%)	Total (*N* = 1,716)	Victimization (*N* = 610; 35.55%)	Perpetration-victimization (*N* = 208; 12.12%)
Technological coping	997 (58.10)	363 (59.51)	126 (60.60)
1. Deleted my profile	447 (26.05)	184 (30.16)	80 (38.46)
2. Changed my settings	331 (19.29)	150 (24.59)	76 (36.54)
3. Deleted from contact list	295 (17.19)	130 (21.31)	64 (30.77)
4. Changed my information	442 (25.76)	185 (30.33)	86 (41.35)
5. Searched for advice	665 (38.75)	229 (37.54)	86 (41.35)
6. Reported to administrator	388 (22.61)	171 (28.03)	85 (40.87)
Reframing	716 (41.72)	292 (47.87)	113 (54.33)
7. Thought to myself not worth my time	374 (21.79)	154 (25.25)	76 (36.54)
8. Thought to myself the person was pitiful	498 (29.02)	206 (33.77)	92 (44.23)
9. Thought to myself that not hurt me	387 (22.55)	185 (30.33)	87 (41.83)
10. Thought to myself nothing serious	469 (27.33)	214 (35.57)	91 (43.75)
Ignoring	697 (40.62)	281 (46.07)	106 (50.96)
11. Decided to ignore it	637 (37.12)	253 (41.48)	93 (44.71)
12. Not to pay attention	624 (36.36)	258 (42.30)	100 (48.08)
Dissociation	1,022 (59.56)	380 (62.30)	130 (62.50)
13. Thought to myself it wasn’t happening in real life	659 (38.40)	263 (43.11)	103 (49.52)
14. Thought to myself the person wouldn’t do it in real life	682 (39.74)	268 (43.93)	105 (50.48)
15. Thought to myself things in real life like that would be much worse	657 (38.29)	235 (38.52)	92 (44.23)
16. Thought to myself things similar happen on the internet	842 (49.07)	317 (51.97)	113 (54.33)
Cognitive avoidance	692 (40.33)	318 (52.13)	119 (57.21)
17. Tried to focus on something else	387 (22.55)	177 (29.10)	82 (39.42)
18. Simply took it lightly	610 (35.55)	281 (46.07)	109 (52.40)
Behavioral avoidance	976 (56.88)	381 (62.46)	139 (66.83)
19. Stopped visiting the webpage	520 (30.30)	228 (37.37)	99 (47.60)
20. Deleted the messages	359 (20.92)	161 (26.39)	79 (37.98)
21. Started avoiding the person in real life	806 (46.97)	293 (48.03)	116 (55.77)
Seeking support	658 (38.24)	254 (41.64)	98 (47.12)
22. Told someone about it	658 (38.34)	254 (41.64)	98 (47.12)
Confrontation	1,056 (61.54)	369(60.49)	124 (59.62)
23. Tried to talk to the person on the internet to persuade him or her to stop	849 (49.48)	298 (48.85)	110 (52.88)
24. Tried face-to-face talking to persuade him or her to stop	951 (55.42)	319 (52.30)	114 (54.81)
Retaliation	1,339 (78.03)	442 (72.46)	132 (63.46)
25. Did something similar face-to-face to that person	1,297 (75.58)	422 (69.18)	125 (60.10)
26. Did something similar online to that person	1,229 (71.62)	373 (61.15)	115 (55.29)

### Regression analyses of coping strategies and cyberbullying experiences

We conducted a series of regression analyses to explore the relationships between coping strategies and cyberbullying experiences in the general adolescent population, as well as SGM. Coping strategies were set as predictors of cyberbullying experience. As shown in Tables 3 and 182 (10.6%) participants reported having experienced cyberbullying victimization, and 124 (7.2%) had experienced both perpetration and victimization, identifying as SGM. In the general adolescent population, cyberbullying victimization was positively related to cognitive avoidance (B = 0.20, *p* < .001). Cyberbullying perpetration-victimization was positively related to reframing (B = 0.06, *p* < .05), seeking support (B = 0.07, *p* < .01), and cognitive avoidance (B = 0.11, *p* < .01). For SGM, cyberbullying victimization was positively related to reframing, cognitive avoidance, and seeking support (Bs ranging from 0.09 to 0.11, all ps < 0.01), and negatively related to retaliation (B = -0.16, *p* < .001). Similar patterns were observed among SGM with experiences of cyberbullying perpetration-victimization (Bs ranging from 0.08 to 0.09, all ps < 0.01). Retaliation was found to be negatively related to both cyberbullying victimization and perpetration-victimization in both the general adolescent population and among SGM (Bs ranging from − 0.14 to -0.19, all ps < 0.001). No significant relationships were found between cyberbullying and other coping strategies in either the general population or among SGM.

**Table 3:** Regression analysis on coping strategies by cyberbullying experiences

Beta	General group		Sexual minorities	
	Victimization	Perpetration-victimization	Victimization	Perpetration-victimization
N (%)	610 (35.55)	208 (12.12)	182 (10.60)	124 (7.22)
Technological	-0.01	-0.01	-0.03	-0.03
Reframing	0.04	0.06*	0.09**	0.08**
Ignoring	-0.03	0.01	-0.01	0.03
Dissociation	-0.03	-0.04	-0.03	-0.04
Cognitive avoidance	0.20***	0.11**	0.11**	0.05
Behavioral avoidance	0.05	0.06*	0.04	0.04
Seeking support	0.04	0.07**	0.10***	0.09***
Confrontation	-0.02	-0.01	0.01	0.01
Retaliation	-0.14***	-0.19***	-0.16***	-0.15***
R^2^	0.06	0.06	0.05	0.04
F	11.16	10.55	9.95	7.95
*p*	< 0.001	< 0.001	< 0.001	< 0.001

*Note* *p < .05. **p < .01. ***p < .001

## Discussion

Our study validated the Chinese version of the CSVC scale, providing evidence of its reliability and validity among Chinese adolescents. This validated scale is poised to be an invaluable tool for future interventions aimed at developing effective coping strategies for potential victims of cyberbullying. It can also be used in school and clinical settings to enhance the coordination of community responses to support both victims and bully-victims.

The CSVC has demonstrated both reliability and internal consistency. The CFA results showed that the eight-factor model fits the data well and exhibits good convergent validity. The Chinese version of the CSVC, which retains the original scale’s eight factors and 25 items, provides a comprehensive tool for screening of coping strategies among Chinese adolescent victims of cyberbullying. The CSVC scale includes specific actions for addressing cyberbullying victimization, such as “stopped visiting the webpage” and “changed my settings.” These actions offer a nuanced perspective for evaluating coping strategies in cyberspace. The findings affirm that the strategies associated with these factors are effectively applicable to Chinese adolescents.

The results of our study indicate that despite retaliation having the highest prevalence rate, there is a negative correlation between experiences of cyberbullying victimization and the likelihood of choosing retaliation. This observation suggests that adolescents who have experienced cyberbullying might regard retaliation as less constructive, thus making them less inclined to engage in such behaviors [23]. Several individual characteristics, such as gender (specifically being male), having higher levels of self-efficacy, immaturity, and perceptions of the strategy’s usefulness, have been previously found to be factors positively related to retaliatory behaviors [5, 34, 35]. Adolescents’ appraisals of the severity of a cyberbullying incident could also influence their decision to confront perpetrators directly, which could make the perpetrators aware of the negative consequences of their actions [36]. However, such direct confrontation is often retrospectively viewed as unhelpful. Cultural factors also play a significant role in shaping coping preferences. For instance, the concept of the “endeavouring self” prevalent in Chinese culture encourages perseverance and overcoming obstacles [37], which may influence the choice of coping strategies. These hypotheses could be further tested in future cross-cultural studies using the same validated measures to examine the effects of cultural orientations on coping strategies, which could provide deeper insights into the dynamics of cyberbullying responses.

Our findings indicate variations in the tendency to seek support between cyberbullying victims and bully-victims. We observed a positive relationship between the tendency to seek support and experiences of perpetration victimization, but no significant relationship was found with victimization alone. This aligns with previous research suggesting that certain perceptions could undermine adolescents’ willingness to seek support, such as susceptibility to peer pressure [13], concerns about jeopardizing their social reputation, and fear of rejection. A previous study showed that adolescents who feel ignored or believe that seeking help is futile may give up seeking help from adults [38]. Some may also hesitate to approach parents or teachers due to worries about losing Internet privileges or receiving unhelpful advice to simply ignore the problem [23]. In the cultural context of China, the “halo effect”, where teachers often have a more favorable view of academically successful students but neglecting those involved in bullying, could discourage students from seeking support, as they perceive informing a teacher to be ineffective [39]. Therefore, in cultures that highly prioritize academic achievement, the emotional and cognitive capacity to seek social support deserves further attention from schools and families. Peer support may also be important in emphasizing psychological well-being in potential victimization.

Both cyberbullying victims and bully-victims within the general adolescent population, as well as SGM, tend to use cognitive avoidance strategies. This finding is consistent with previous research indicating that cyberbullying victims are more likely to adopt avoidance coping strategies compared to adolescents who have not experienced cyberbullying [40]. Victims often evaluate cyberbullying as unsolvable, feel helpless, and believe they have no choice but to avoid thinking about the trauma [4]. In a collectivist cultural context, avoidance may also pertain to saving one’s face and that of significant others to maintain harmony [5]. Adherence to the value of filial piety may lead victims to refrain from talking about victimization to spare parents’ worry. SGM adolescents, in particular, may avoid reporting bullying victimization due to fears of involuntary disclosure of their sexual identity by school staff to other students and family members [2]. Although having higher levels of social support within their sexual identity groups, SGM victims often perceive considerably less support from broader society [2]. We also found that SGM victims preferred reframing as a coping strategy, which involves re-evaluating or reconstructing situations positively and attributing the perpetrators’ negative behaviors to situational factors, thereby reducing their own emotional distress [2]. Our findings reveal different patterns of coping strategies for cyberbullying among victims of various orientations. These patterns can be tested in future studies using diverse groups that employing equivalent measures.

### Limitations

This study has several limitations. First, our findings are based on cross-sectional data from respondents in two newly designated first-tier cities in China. Adolescents in these districts come from economically above-average economic status. Given the significant digital divide in China, this sample may not accurately represent the broader spectrum of adolescents across different socioeconomic backgrounds. This limits the generalizability of our results. Future research should apply this scale to a more diverse group of respondents and adopt more culturally adapted items to assess coping strategies among Chinese adolescents. Second, the participants were asked to report their responses to perceived cyberbullying behaviors, which may not be consistent with their actual strategies in real-life incidents. Future studies should collect data from victims who are actively seeking help from school professionals for cyberbullying. This approach would allow for the recording of actual behaviors and for comparing the effectiveness of the coping strategies used. Third, the potential for social desirability bias for sexual orientation in East Asian cultures may have contributed to underreporting. Alternative approaches to self-report designs should be considered in future research and practice to explore the well-being of SGM more accurately, as well as other marginalized groups of adolescents.

### Implications

Despite these limitations, our study contributes to the field of cyberbullying victimization by introducing a robustly validated scale. Researchers can employ this measure for cross-cultural comparisons among adolescents in different countries and districts. Direct comparisons of the effectiveness of cyber-specific strategies with those of traditional strategies should also be considered in future studies. For example, previous studies have revealed that limiting one’s Internet use and blocking contacts are associated with decreased victimization, while strategies such as retaliation and avoidant behavior have been found to be ineffective [12]. Future studies should examine the mechanisms that lead from victimization to the adoption of different coping strategies.

Schools should implement comprehensive programs that educate students, teachers, and staff about the nature of cyberbullying, its consequences, and the importance of reporting incidents. The CSVC scale can be used to tailor these programs by highlighting coping strategies that are effective in the Chinese cultural context. Schools and social service centers can use the CSVC scale to guide future individualized interventions aimed at combating cyberbullying. The inclusion of SGM adolescents highlights the need to formulate measures that differ from those used by the general population when faced with cyberbullying. Establish a peer mentoring system where older students trained in the use of the CSVC scale can also support younger students, fostering a community of care and vigilance against cyberbullying especially for those SGM victims.

Importantly, the perceived control of parents can affect the selection and effectiveness of certain coping strategies. It is crucial for schools to strengthen educate families to respond to children seeking support in a timely and effective manner. For example, offer workshops that teach parents about the dynamics of cyberbullying and effective coping strategies identified by the CSVC scale. These workshops should provide practical advice on how to communicate with children about their online experiences and how to support them in using effective coping mechanisms. Parenting programs are suggested to promote parent-child interactions, which can aid children in identifying and resolving online conflicts and responding with positive coping strategies, both as victims and as bystanders. In this manner, the CSVC scale can serve as a resource for identifying which strategies might be most suitable for their children.

## Conclusions

In conclusion, the validation of the Chinese version of the Coping Strategies for Victims of Cyberbullying (CSVC) scale offers invaluable insights for addressing cyberbullying amongst adolescents. By providing a culturally sensitive tool to assess and understand coping mechanisms, this research extends its significance beyond theoretical bounds and into the realm of practical application. The detailed recommendations proposed for schools and families are poised to transform the landscape of cyberbullying intervention strategies. Through the implementation of targeted educational programs, the establishment of peer support systems, and the creation of comprehensive crisis protocols, schools can become safer environments where students are equipped to handle cyberbullying effectively. The CSVC scale stands as a cornerstone for these initiatives, guiding the development of tailored interventions that resonate with the needs of Chinese adolescents. It is imperative that the momentum generated by this research is harnessed to foster resilience among adolescents, ensuring they are not only protected but also empowered to navigate the challenges of the cyber world.

### Electronic supplementary material

Below is the link to the electronic supplementary material.

Supplementary Material 1

## Data Availability

The datasets used and/or analysed during the current study are available from the corresponding author on reasonable request.
