# Supplementary material for: Validation of the Chinese version of the coping strategies for victims of cyberbullying scale
Source: BMC Psychol. 2024 May 10;12:259. doi: 10.1186/s40359-024-01766-x (PMC11084028; doi:10.1186/s40359-024-01766-x)
Supplement: Supplementary file 1 — Supplementary Material 1 [file 40359_2024_1766_MOESM1_ESM.docx]

Table S1. Summary table of eight-factor construct correlation matrices and squared correlation coefficients

|  | F1 | F2 | F3 | F4 | F5 | F6 | F7 | F8 |  |
| --- | --- | --- | --- | --- | --- | --- | --- | --- | --- |
| F1 | 0.47 |  |  |  |  |  |  |  |  |
| F2 | 0.19 | 0.56 |  |  |  |  |  |  |  |
| F3 | 0.10 | 0.63 | 0.84 |  |  |  |  |  |  |
| F4 | 0.13 | 0.43 | 0.46 | 0.62 |  |  |  |  |  |
| F5 | 0.24 | 0.63 | 0.67 | 0.65 | 0.49 |  |  |  |  |
| F6 | 0.37 | 0.35 | 0.32 | 0.43 | 0.89 | 0.46 |  |  |  |
| F7 | 0.08 | 0.11 | 0.07 | 0.17 | 0.18 | 0.20 | 0.65 |  |  |
| F8 | 0.02 | 0.04 | 0.05 | 0.10 | 0.05 | 0.06 | 0.29 | 0.78 |  |
|  |  |  |  |  |  |  |  |  |  |

Note. the diagonal values are Average variation extraction (AVE) value; The upper triangle values are the squared correlation coefficients between the factors.
